# Effect of Obesity on Mortality and Morbidity After Coronary Artery Bypass Grafting Surgery in Iranian Patients

**DOI:** 10.5812/aapm.18884

**Published:** 2014-05-04

**Authors:** Maryam Ardeshiri, Zahra Faritous, Zahra Ojaghi Haghighi, Shirin Hosseini, Ramin Baghaei

**Affiliations:** 1 Rajaie Cardiovascular Medical and Research Center, Iran University of Medical Sciences, Tehran, Iran

**Keywords:** Obesity, Body Mass Index, Coronary Artery Bypass, Complications, Mortality

## Abstract

**Background:**

Recent years have witnessed the emergence of obesity as a major public health concern. The drastic rise in obesity and its concomitant co-morbidities is a reflection of the recent changes in dietary habits in Iran and many other developing countries. A recent large population study in Tehran reported that 58% and 75% of middle-aged Iranian men and women, respectively, were either overweight or obese.

**Objectives:**

Considering the impact of obesity on mortality and morbidity after coronary artery bypass graft surgery (CABG), we sought to investigate the association between central obesity and the body mass index (BMI) and the post-CABG mortality and morbidity in Iranian patients.

**Patients and Methods:**

This prospective study was on 235 adult patients scheduled for isolated CABG in a university hospital. The patients were divided in two groups according to BMI ≥ 30 (obese; n = 60) and BMI < 30 (non-obese; n = 175). In-hospital and late (after 3 months) morbidity and mortality rates were compared between obese and non-obese patients.

**Results:**

A total of 235 patients (135 women) with a mean age of 59 ± 9.2 years (range = 29 to 79 years), mean BMI of 27.3 ± 4.2 (range = 17 to 40), and mean waist circumference of 101.2 ± 14.7 cm (range = 55 to 145 cm) were included. By the third postoperative month, wound infection had significantly increased in patients with BMI ≥ 30 (P = 0.022). In-hospital and late morbidity and mortality rates were comparable between the two groups (P > 0.05).

**Conclusions:**

In our patients obesity was a risk factor for wound infection but not atelectasis or the need for intra-aortic balloon pump or re-exploration. Obesity was not associated with increased in-hospital or 3 months mortality rates after CABG.

## 1. Background

Recent years have witnessed the emergence of obesity as a major public health concern. The drastic rise in obesity and its concomitant co-morbidities is a reflection of the recent changes in dietary habits in Iran (1) and many other developing countries (2). A recent large population study in Tehran reported that 58% and 75% of middle-aged Iranian men and women, respectively, were either overweight or obese (3). Obesity can promote the atherosclerotic process by disrupting the endothelial function as well as enhancing oxidative stress and induction of pro-inflammatory states (4, 5).

## 2. Objectives

Obesity is, therefore, regarded as a risk factor for mortality and morbidity after coronary artery bypass graft surgery (CABG) (6-9). There is a relatively wide divergence of opinions on the relationship between the body mass index (BMI) and post-CABG morbidity and mortality in the current literature: There is a school of thought maintaining that BMI is not a risk factor for mortality (10, 11), whilst some other investigators consider obesity as a protective factor in patients undergoing CABG (12). What most experts agree on its relation with post-CABG complications, however, is waist circumference (WC), which is deemed the best and most simple anthropometric measurement for central obesity. Yet no previous study has hitherto evaluated the effect of obesity, in terms of WC, on post-CABG morbidity and mortality in Iran. We, therefore, aimed to investigate the association between obesity (BMI) and mortality and morbidity rates in Iranian patients following CABG.

## 3. Patients and Methods

### 3.1. Study Population

Between March, 2010 and February, 2011, 235 patients with age over 18 years, and coronary artery disease who were referred to a university cardiovascular and research center for isolated CABG were investigated. The institutional approval number was 88.28. This is an analytic study. In all patients with American Society of Anesthesiologists (ASA) physical status class II or III, preoperative and operative data were prospectively collected and entered into a computerized database. The anaesthetic and surgical techniques were standardized for all of the patients. Anesthesia was induced using midazolam or propofol plus sufentanil and cisatracuriumor atracurium, followed by infusion dose of these drugs. Midline sternotomy, hypothermia 30-32 °C, and antegrade cold blood cardioplegia were used. Demographic and baseline data, clinical information, and ICU sheets in addition to data on risk factors, medication, and functional status were obtained by trained personnel supervised by a research nurse. Postoperative complications were also recorded prospectively by a researcher, and all major adverse events were prospectively validated by an experienced cardiac surgeon according to standard clinical definitions. All the patients were followed up in hospital and for a three-month follow-up period; follow-up was completed by all the patients. The exclusion criteria included: age < 18 years, severe co-morbidities, history of valvular heart disease, concomitant procedures, and low left ventricular ejection fraction (LVEF < 35%) on admission echocardiography. This study was approved by the institutional ethics committee and informed consent was obtained from all patients. This study was not been financially supported by any funding resource. 

### 3.2. Clinical, Anthropometrical, and Laboratory Measurements

The patients’ characteristics, including age, sex, history of diabetes mellitus (DM), hypertension, hyperlipidemia, smoking, BMI, waist circumference, fasting blood glucose and cholesterol and triglyceride levels, were collected. N number and sites of the grafts were also recorded.

### 3.3. Definition of Obesity

BMI was measured as weight in kilograms divided by height in square meters (Kg/m^2^). Patients with BMI ≥30 Kg/m^2^ were classified as obese and those with BMI < 30 Kg/m^2^ considered as non-obese. WC was measured by placing a tape measure horizontally at the level of the iliac crest in non-stress situation. WC ≥102 cm in men and 88 cm in women was considered as obesity, and WC < 102 cm in men and < 88 cm in women was defined as non-obesity (13).

### 3.4. Outcomes Follow Up

The primary end-point of our study was operative mortality, defined as death from any cause occurred in-hospital and within three months after surgery. Cardiac and non-cardiac morbidities constituted the secondary end-points, which included arrhythmias, sepsis, heart failure cardiogenic shock (systolic blood pressure < 90 mmHg with no or poor response to fluids and requiring administration of inotropic infusions to maintain blood pressure and requirement for an intra-aortic balloon pump), aortic cross-clamp time atelectasia, re-intubation, renal failure, intra-aortic balloon pump requirement, bleeding, wound infection, lung infection, re-exploration, multi organ failure, tamponade, myocardial infarction, bedsore, pulmonary embolism, mechanical ventilation time (hour), ICU stay time (day) and re-admission due to mediastinitis. The criterion for postoperative sepsis was the occurrence of a positive blood culture. The criterion for lung infection was a positive culture of the sputum plus a radiological infiltration. The criterion for postoperative renal failure was a 50% increase in the baseline serum creatinin level. Multiorgan failure was defined as involvment of two or more organ systems. Mediastinitis was diagnosed when a deep sternal infection was present, necessitating exploration of the wound with excision of tissues and treatment with antibiotics. Mortality was defined as in-hospital death and death within a three-month period after the operation. All the follow-up data were collected when the patient was discharged for a period of three months following CABG. Follow-up was completed by all patients.

### 3.5. Statistical Analysis

The data are described as mean ± standard deviation (SD) for interval and count (%) for categorical variables. We used the chi-square test for categorical variables and student’s t test for numerical variables. P value ≤ 0.05 was considered as statistically significant result. SPSS^®^ 15.0 for Windows^®^ (SPSS Inc. Chicago, Illinois) was used for statistical analyses.

## 4. Results

### 4.1. Background Data

A total of 235 patients (135 women) with a mean age of 59 ± 9.2 years (range = 29 to 79 years), mean BMI of 27.3 ± 4.2 kg/m^2^ (range = 17 to 40 kg/m^2^), and mean WC of 101.2 ± 14.7 cm (range = 55 to 145 cm) were recruited in the present study. Table 1 depicts the descriptive data on the study population’s background and demographics in obese and non-obese patients, and Table 2 illustrates the postoperative outcomes compared between the two groups.The mean age in patients with BMI ≥30 kg/m^2^ (59 ± 8.7 kg/m^2^) was lower than that in patients with BMI < 30 kg/m^2^ (60 ± 9.5 kg/m^2^), but this difference was not statistically significant. Those with BMI ≥30 were more likely to be female (P < 0.001) and have diabetes mellitus (P = 0.033). Total serum cholesterol (P < 0.001), serum low-density lipoprotein (P < 0.001), serum triglyceride (P = 0.016), WC (P < 0.001), and ejection fraction (P = 0.037) were significantly higher in the obese patients (BMI ≥ 30), whilst smoking was more frequent in those with BMI < 30 (P = 0.027). There was no difference between the site of coronary involvement and obesity when patients were startified according to their BMI. Eight 8 (57.1%) of male patients and 30 (65.2%) of female patients with BMI ≥ 30 had DM (P = 0.004), and 7 (23.3%) of male patients and 23 (76.7%) of female patients with BMI ≥ 30 had dyslipidemia (P = 0.034; Table 3).

**Table 1.** The General Characteristics of Patients According to Body Mass Index (BMI) ^a^

Characteristic / Variable	Total (n = 235)	BMI < 30 (n = 175)	BMI ≥ 30 (n = 60)	P value
**Age, y**	59 ± 9.2	60 ± 9.5	59 ± 8.7	0.386
**Gender, Female/Male**	135/100	89/86	(6/14)	< 0.001 ^b^
**Diabetes mellitus**	121 (51.5)	83 (47.4)	38 (63.3)	0.033 ^b^
**Hyperlipidemia (history)**	107 (45.5)	77 (44)	30 (50)	0.421
**Hypertension**	136 (57.9)	99 (56.6)	37 (61.7)	0.49
**Waist Circumference, cm**	101.2 ± 14.7	97.9 ± 14.2	110.8 ± 11.7	< 0.001 ^b^
**Addiction**	28 (12)	24 (13.8)	4 (6.7)	0.141
**Alcohol**	1 (0.4)	1 (0.6)	0	0.557
**Smoking**	20 (8.5)	19 (10.9)	1 (1.7)	0.027 ^b^
**Ejection Fraction, %**	45.2 ± 9.5	44.5 ± 9.6	47.5 ± 9	0.037 ^b^
**Para-Clinical Finding**				
Fasting Serum Glucose, mg/dl	145 ± 60.4	142 ± 60.1	153.82 ± 61	0.085
Total Serum Cholesterol, mg/dl	174.3 ± 53.8	166.8 ± 49.6	196.1 ± 59.8	< 0.001 ^b^
Serum High Density Lipoprotein, mg/dl	39.8 ± 54.9	41.2 ± 63.3	35.8 ± 11	0.877
Serum Low Density Lipoprotein, mg/dl	90.2 ± 37.4	85.7 ± 37.6	103.4 ± 33.8	<0.001 ^b^
Serum Triglyceride, mg/dl	201.1 ± 142.8	186.7 ± 105.2	243.3 ± 214	0.016 ^b^
Serum Creatinin, mg/dl	1.3 ± 0.3	1.3 ± 0.3	1.2 ± 0.3	0.086
**Involved Coronary Artery**				
Left Main	6 (2.6)	6 (3.4)	0	0.15
Left Anterior Descending	234 (99.6)	174 (99.4)	60 (100)	0.557
Left Circumflex	203 (86.4)	155 (88.6)	48 (80)	0.095
Right Coronary Artery	202 (86)	152 (86.9)	50 (83.3)	0.498
Total graft number	3.3 ± 0.9	3.3 ± 0.9	3.1 ± 0.9	0.048 ^b^
Cardiopulmonary bypass time, min	100.2 ± 55.3	101.9 ± 56.9	95.5 ± 50.5	0.356
Cross-clamp time, min	56.4 ± 42.9	56.2 ± 44.3	57 ± 39	0.499
Mechanical ventilation time, h	25.1 ± 89.5	27.2 ± 103.4	19 ± 12.8	0.589

^a^ Data are presented as Mean ± SD and No. (%).

^b^ Significant P value.

### 4.2. Early and Late Complications

The incidence of complications after three months was low. The only outcome which was significantly different among the BMI categories was wound infection (at three months’ follow-up) (P = 0.022), whilst three (5%) of the patients with BMI ≥ 30 kg/m^2^ had this complication at three months’ follow-up. Detailed outcomes, with regard to the BMI, are listed in Tables 2 and 3. Adjusted associations between the different outcomes and BMI ≥ 30, history of diabetes and sex were determined by logistic regression models; there was no statistically significant association between them. All the patients who died had central obesity, but the p value was not significant.

**Table 2.** Post-Operative Outcomes According to Body Mass Index (BMI) ^a^

Characteristic / Variable	Total	Body Mass Index < 30 (n = 175)	Body Mass Index ≥ 30 (n = 60)	P value
**Arrhythmia**	18 (7.7)	16 (9.1)	2 (3.3)	0.144
**Sepsis**	3 (1.3)	3 (1.7)	0	0.307
**Congestive heart failure**	2 (0.9)	1 (0.6)	1 (1.7)	0.425
**Cardiac shock**	2 (0.9)	2 (1.1)	0	0.406
**Atelectasia**	16 (6.8)	12 (6.9)	4 (6.7)	0.961
**Reintubation**	3 (1.3)	2 (1.1)	1 (1.7)	0.755
**Renal failure**	2 (0.9)	2 (1.1)	0	0.406
**Intra-aortic balloon Pump**	9 (3.8)	8 (4.6)	1 (1.7)	0.312
**Bleeding**	20 (8.5)	16 (9.1)	4 (6.7)	0.553
**Wound infection**	9 (3.8)	8 (4.6)	1 (1.7)	0.312
**In-hospital death**	3 (1.3)	3 (1.7)	1 (1.7)	0.982
**Re-exploration**	8 (3.4)	6 (3.4)	2 (3.3)	0.972
**Lung infection**	4 (1.7)	4 (2.3)	0	0.238
**Multi-organ failure**	0	0	0	-
**Tamponade**	3 (1.3)	2 (1.1)	1 (1.7)	0.755
**Myocardial infarction**	12 (5.1)	10 (5.7)	2 (3.3)	0.473
**Bedsore**	7 (3)	5 (2.9)	2 (3.3)	0.851
**Pulmonary embolism**	3 (1.3)	2 (1.1)	1 (1.7)	0.755
**ICU stay time, day ** ^**b**^	4.9 (3-8)	5.2 (3-10)	4± (3-6)	0.542
**Sepsis, after 3 months**	0	0	0	-
**Cardiac Shock, after 3 months**	1 (0.4)	1 (0.6)	0	0.557
**Wound Infection, after 3 months**	4 (1.7)	1 (0.6)	3 (5)	0.022 ^c^
**Death, after 3 months**	4 (1.4)	2 (1.1)	1 (1.7)	0.755
**Lung Infection, after 3 months**	2 (0.9)	1 (0.6)	1 (1.7)	0.425
**Myocardial Infarction, after 3 months**	3 (1.3)	2 (1.1)	1 (1.7)	0.755
**Pulmonary Emboli, after 3 months**	8 (3.4)	7 (4)	1 (1.7)	0.391
**Re-admission, after 3 months**	24 (10.2)	18 (10.3)	6 (10)	0.952
**Mediastinitis, after 3 months**	9 (3.8)	7 (4)	2 (3.3)	0.816

^a^ Data are presented as No. (%).

^b^ Median (Interquartile range).

^c^ Significant P value.

**Table 3.** Post-Operative Outcomes According to BMI in Both Sexes ^a^

Characteristic/ Variable	BMI < 30		BMI ≥ 30		P value ^b^
	Women = 89	Men = 86	Women = 46	Men = 14	
**Diabetes**	52 (58.4)	31 (36)	30 (65.2)	8 (57.1)	0.004^b^
**Hyperlipidemia (History)**	47 (61)	30 (39)	23 (76.7)	7 (23.3)	0.034^b^
**HDL-Dyslipidemia ** ^**c**^	75 (84.3)	65 (75.6)	42 (91.3)	9 (64.3)	0.025^b^
**Arrhythmia**	10 (11.2)	6 (7)	2 (4.3)	0	0.244
**Sepsis**	1 (1.1)	2 (2.3)	0	0	0.549
**Congestive heart failure**	0	1 (1.2)	0	1 (7.1)	-
**Cardiac Shock**	2 (2.2)	0	0	0	-
**Atelectasia**	8 (9)	4 (4.7)	4 (8.7)	0	0.132
**Re-intubation**	0	2 (2.3)	1 (2.2)	0	0.32
**Renal Failure**	2 (2.2)	0	0	0	-
**Intra-Aortic balloon Pump**	4 (4.5)	4 (4.7)	0	1	0.58
**Bleeding**	7 (7.9)	9 (10.5)	3 (6.5)	1 (7.1)	0.561
**Wound Infection**	4 (4.5)	4 (4.7)	1 (2.2)	0	0.908
**In-hospital death**	2 (2.2)	1 (1.2)	1 (2.2)	0	0.463
**Re-exploration**	3 (3.4)	3 (3.5)	1 (2.2)	1 (7.1)	0.672
**Lung Infection**	2 (2.2)	2 (2.3)	0	0	0.972
**Multi organ failure**	0	0	0	0	-
**Tamponade**	2 (2.2)	0	1 (2.2)	0	-
**Myocardial Infarction**	4 (4.5)	6 (7)	2 (4.3)	0	0.701
**Bedsore**	5 (5.6)	0	1 (2.2)	1 (7.1)	0.2
**Pulmonary Embolism**	2 (2.2)	0	1 (2.2)	0	-
**ICU stay time, day** ^**d**^	5.5 (3-11)	4.9 (3-10)	4 (3-8)	4 (3-9)	0.823
**Cardiopulmonary bypass time, min**	95.1 ± 48.2	109 ± 64.2	89.9 ± 46.1	113.7 ± 61.2	0.598
**Cross Clamp Time, min**	50.6 ± 35.5	61.9 ± 51.5	54.3 ± 37.1	65.6 ± 44.9	0.123
**Mechanical ventilation time, h**	32.4 ± 143.2	21.8 ± 24.8	17.8 ± 9.5	23.1 ± 20.3	0.603
**Sepsis, after 3 months**	0	0	0	0	-
**Cardiac Shock, after 3 months**	0	1 (1.2)	0	0	-
**Wound Infection, after 3 months**	1 (1.1)	0	3 (6.5)	0	-
**Death, after 3 months**	1 (1.1)	1 (1.2)	1 (2.2)	0	0.785
**Lung Infection, after 3 months**	0	1 (1.2)	1 (2.2)	0	0.643
**Myocardial Infarction, after 3 months**	2 (2.2)	0	1 (2.2)	0	-
**Pericardial Effusion, after 3 months**	2 (2.2)	5 (5.8)	1 (2.2)	0	0.329
**Re-admission, after 3 months**	9 (10.1)	9 (10.5)	6 (13)	0	0.57
**Mediastinitis, after 3 months**	5 (5.6)	2 (2.3)	2 (4.3)	0	0.192

^a^ Data are presented as Mean ± SD and No. (%)

^b^ P value for interaction between BMI and sex.

^c^ HDL < 40 mg/dl for men; HDL < 50 mg/dl for women.

^d^ Median(Interquartile range).

## 5. Discussion

Our study showed a high prevalence of obesity in coronary artery disease patients scheduled for CABG. The prevalence of obesity in our patients, as indicated by BMI, was 25.5%, which is in agreement with some previous studies. However, it has been posited that WC is a stronger predictor of cardiovascular disease outcomes than BMI (14). Recent studies have demonstrated a dramatic rise(as high as 35%) in the prevalence of obesity in the Iranian population and Asian countries (15). It is worth noting that the number of obese patients undergoing CABG has also been on the increase in recent years (16). Intra-abdominal fat is resistant to insulin, giving rise to hyperlipidemia, glucose intolerance, hypertension and atherosclerosis. These conditions, in turn, induce adipokinase and enhance chronic low-grade inflammation (17). Consistent with the findings of some previous studies, females accounted for the majority of our obese patients (8). The frequency of diabetes and hyperlipidemia was also high in female patients in comparison with their male counterparts. In contrary, some investigators have suggested that obesity is a protective factor in patients undergoing CABG (18-20). The wound infection rates at three months’ follow-up post-CABG were high in our obese patients in comparison with our non-obese patients, which is in agreement with the findings of some previous studies (21-24). Some studies have not found any difference between obese and non-obese patients in terms of morbidity (25-27) and mortality (10, 11, 28). But others have demonstrated that the rates of ventricular tachycardia 10, myocardial infarction, hospital admission times (10, 24, 29) and atrial fibrillation (24) are significantly lower in obese patients. In our study, there was no association between obesity and mortality. However, some investigations have reported a significant difference regarding mortality between obese and non-obese patients undergoing CABG (29, 30). The impact of obesity on mortality may have decreased over time and it is thought to be related to the decline in the prevalence of cardiovascular risk factors and improvements in public health and medical care (29). This discrepancy in the aforementioned findings may be due to the differences in the definitions of obesity, cut-off points for group classification, study durations, and patient selection criteria. Moreover, what can also impact the outcome of a study is whether the study population is composed of emergent or non-emergent patients or whether some other surgical procedures are performed in tandem with CABG. The short follow-up duration of the present study is its most significant limitation. Studies with longer follow-up durations are required to shed further light on this issue. In our patients obesity was a risk factor for wound infection but not atelectasis, intra-aortic balloon pump or re-exploration. In this study obesity did not predict increased in-hospital or three months mortality rates after CABG. Patients with central obesity have more established coronary artery disease and CABG may be necessary for many obese persons. Therefore, they benefit from early aggressive risk reduction. It is, therefore, advisable that obesity (especially central obesity) in patients scheduled for CABG be identified and managed aggressively in order to reduce morbidity.
